# Prioritizing rural populations in state comprehensive cancer control plans: a qualitative assessment

**DOI:** 10.1007/s10552-023-01673-3

**Published:** 2023-02-25

**Authors:** Rachel Hirschey, Catherine Rohweder, Whitney E. Zahnd, Jan M. Eberth, Prajakta Adsul, Yue Guan, Katherine A. Yeager, Heidi Haines, Paige E. Farris, Jennifer W. Bea, Andrea Dwyer, Purnima Madhivanan, Radhika Ranganathan, Aaron T. Seaman, Thuy Vu, Karen Wickersham, Maihan Vu, Randall Teal, Kara Giannone, Alison Hilton, Allison Cole, Jessica Y. Islam, Natoshia Askelson

**Affiliations:** 1 School of Nursing, UNC Chapel Hill, and Lineberger Comprehensive Cancer Center, Chapel Hill, NC USA; 2 Center for Health Promotion & Disease Prevention, UNC Chapel Hill, Chapel Hill, NC USA; 3 Department of Health Management and Policy, College of Public Health, University of Iowa, Iowa City, IA USA; 4 Department of Health Management and Policy, Dornsife School of Public Health, Drexel University, Philadelphia, PA USA; 5 Department of Internal Medicine, University of New Mexico & University of New Mexico Comprehensive Cancer Center, Albuquerque, NM USA; 6 Rollins School of Public Health, Emory University, Atlanta, GA USA; 7 Nell Hodgson School of Nursing, Emory University, Atlanta, GA USA; 8 Prevention Research Center for Rural Health, College of Public Health, University of Iowa, Iowa City, IA USA; 9 Knight Cancer Institute, Oregon Health and Science University, Portland, OR USA; 10 Department of Health Promotion Sciences, Mel & Enid Zuckerman, College of Public Health, University of Arizona, Tucson, AZ USA; 11 Community and Behavioral Health, The Colorado School of Public Health, University of Colorado, Aurora, CO USA; 12 University of Arizona Mel & Enid Zuckerman College of Public Health, Tucson, AZ USA; 13 Department of Epidemiology and Biostatistics, Arnold School of Public Health, University of South Carolina, Columbia, SC USA; 14 Department of Internal Medicine, Carver College of Medicine, University of Iowa, Iowa City, IA USA; 15 MPH Health Promotion Research Center, University of Washington, Seattle, WA USA; 16 College of Nursing, University of South Carolina, Columbia, SC USA; 17 UNC CHAI Core, Connected Health Applications & Interventions (CHAI) Core, Lineberger Comprehensive Cancer Center, Chapel Hill, NC USA; 18 University of Washington, Seattle, WA USA; 19 Moffitt Cancer Center, University of South Florida, Tampa, FL USA; 20 Department of Community & Behavioral Health, College of Public Health, University of Iowa, Iowa City, IA USA

**Keywords:** Comprehensive cancer control, Rural health, Health disparities, Cancer

## Abstract

**Purpose:**

The Centers for Disease Control and Prevention’s National Comprehensive Cancer Control Program (NCCCP) requires that states develop comprehensive cancer control (CCC) plans and recommends that disparities related to rural residence are addressed in these plans. The objective of this study was to explore rural partner engagement and describe effective strategies for incorporating a rural focus in CCC plans.

**Methods:**

States were selected for inclusion using stratified sampling based on state rurality and region. State cancer control leaders were interviewed about facilitators and barriers to engaging rural partners and strategies for prioritizing rural populations. Content analysis was conducted to identify themes across states.

**Results:**

Interviews (*n* = 30) revealed themes in three domains related to rural inclusion in CCC plans. The first domain (barriers) included (1) designing CCC plans to be broad, (2) defining “rural populations,” and (3) geographic distance. The second domain (successful strategies) included (1) collaborating with rural healthcare systems, (2) recruiting rural constituents, (3) leveraging rural community–academic partnerships, and (4) working jointly with Native nations. The third domain (strategies for future plan development) included (1) building relationships with rural communities, (2) engaging rural constituents in planning, (3) developing a better understanding of rural needs, and (4) considering resources for addressing rural disparities.

**Conclusion:**

Significant relationship building with rural communities, resource provision, and successful strategies used by others may improve inclusion of rural needs in state comprehensive cancer control plans and ultimately help plan developers directly address rural cancer health disparities.

**Supplementary Information:**

The online version contains supplementary material available at 10.1007/s10552-023-01673-3.

## Introduction

The National Comprehensive Cancer Control Program (NCCCP) funded by the Centers for Disease Control and Prevention (CDC) provides guidance, funding, and technical assistance to support states, territories, and Native nation organizations in their cancer prevention and control efforts. The CDC requires these entities to develop a Comprehensive Cancer Control (CCC) plan every five years [1]. The CDC’s Self-Assessment Tool [2] provides guidance for elements, such as data on cancer burden and disparities (including geographic disparities), as well as goals, objectives, and strategies to address areas along the cancer continuum and CDC/NCCCP priority areas [2]. Further, the Self-Assessment Tool contains recommendations for engaging diverse partners as part of the planning, decision-making, evaluation, and implementation of CCC plans. However, application of the Self-Assessment Tool varies among states, with significant differences in CCC plan structure and content. These variations cause some elements, such as attention to rural needs, to be inadequately addressed across all state CCC plans.

Rural populations have a disproportionate prevalence of some cancer-relevant risk factors and cancer burden across the cancer continuum from prevention through mortality. These include higher rates of smoking, lower rates of human papillomavirus (HPV) vaccination, lower rates of cancer screening, higher rates of preventable cancers (e.g., cervix, lung, colorectal), and higher rates of cancer mortality compared to their urban counterparts [3–9]. Rural/urban disparities may be due, in part, to higher rates of uninsured individuals and less access to both primary and specialty care [10–12]. More consistent prioritization of rural needs in CCC plans is warranted to achieve equity in cancer prevention, detection, and treatment between rural and urban residents.

In 2019, the National Advisory Committee on Rural Health and Human Services (NACRHHS) released a report with recommendations to address rural cancer prevention and control [13]. One recommendation was for the CDC to require state jurisdictions to assess rural cancer mortality burden and develop goals, objectives, and strategies to address disparities through their CCC plans [13]. A recent assessment of state CCC plans found that over two-thirds included the term “rural,” but only about a third included rural-specific goals, objectives, or strategies [14]. Engaging rural partners in the planning process may facilitate the inclusion of data on rural cancer burden and corresponding goals and objectives to reduce disparities, in accordance with recommendations from NACRHHS and the CDC’s Self-Assessment Tool [2, 13]. However, the extent to which CCC plan developers and their respective cancer coalitions engage with rural partners to address rural cancer disparities in their plans is unknown. Therefore, we conducted a qualitative study to examine how rural perspectives and needs are prioritized in state CCC plans.

## Methods

This study was conducted by the Cancer Prevention and Control Research Network (CPCRN), a CDC-funded research network of eight universities across the USA. The mission of the CPCRN, founded in 2002, is to accelerate the uptake of evidence-based cancer prevention and control strategies to benefit populations that are disproportionately impacted by cancer [15]. Members of the CPCRN rural cancer workgroup conducted 30 interviews with state cancer control leaders to assess how they engage rural partners and address rural priorities in their CCC plans. Interviews were conducted between May and November 2021 by interviewer teams from seven CPCRN centers and a CPCRN affiliate member from Oregon Health & Science University. Institutional Review Board approval was received from the University of Iowa, the University of South Carolina, the University of New Mexico, Emory University, the University of Colorado, the University of North Carolina, the University of Washington, and Oregon Health & Science University.

### Sampling and recruitment

States were identified for study inclusion using stratified random sampling by rurality and region. First, each of the 50 states was categorized as “high” (above average) or “low” (average/below average) rurality. Because 20.4% of the U.S. population lives in rural areas [16], “high” rural states were defined as those in which more than 20.4% of the population lives in rural (non-metropolitan) counties; “low” rural states were defined as those in which 20.4% or less of the population lives in rural counties. Second, the region of each of the 50 states was identified as Western, Midwestern, Southern, or Northeastern, based on the U.S. Census. After this categorization, fifteen “high” rural and fifteen “low” rural states were proportionally selected based on regional distribution. This approach was used to capture a range of possible rural contexts across CCC plans. State cancer control leaders from the selected states were emailed a study overview and instructions for interested parties to contact the research team to schedule an interview. Leaders from some states requested that a colleague join them in the interview or complete the interview in their place. When recruitment emails were not returned or individuals declined participation, another state was randomly selected using the same stratification approach with non-replacement of the declining state. Ultimately, 38 states were contacted to achieve target enrollment of 30 states.

### In-depth interviews

Interviews were conducted by CPCRN rural cancer workgroup researchers. Consistency across interviews was supported by interviewer training and a structured interview guide (Online Appendix: Standard Interview Guide). The guide was created by the research team and contained items to assess the inclusion of “rural” in CCC plan development, content, implementation, evaluation, and future plans. The guide was pilot tested with two individuals from state cancer consortia who were familiar with their CCC plans. Interviews were recorded via video conferencing platforms and lasted between 45 and 60 min. Participants who completed interviews were offered a $30 gift card, although some states had restrictions on employee ability to accept payment.

### Analysis

Audio recordings were transcribed and imported into Dedoose [17] to facilitate analysis. Transcripts were coded using a codebook based on the research questions, interview questions, and notes taken during data collection. The initial codebook was used by four qualitative researchers (MV, RT, KG, AH) to independently code several transcripts. Codes were compared across the four researchers, and each discrepancy was identified and reconciled through discussion and consensus. This process continued until replicability of coding occurred across researchers, at which point the codes and coding decisions were finalized. The final version of the codebook (Online Appendix: Qualitative Analysis Codebook) was then applied, by one researcher per transcript, to the remaining transcripts. The researchers generated reports for each code to create narrative summaries, identify emergent themes and sub-themes, and select illustrative quotes. Subsequently, two other qualitative researchers (RH, CR) applied conventional content analysis [18] to the narrative summaries to identify the three domains and corresponding themes detailed in this paper. The goal of the analytic approach was to synthesize findings across themes rather than conduct a direct comparison between respondents from “high” rural versus “low” rural states.

## Results

The final sample (*n* = 30) included eighteen “high” rural states and twelve “low” rural states; four were from the Northeast, nine from the South, eight from the Midwest, and nine from the West. The states included in this study are not specified to protect participant confidentiality, as state cancer control directors are easily identifiable. Two additional state cancer control leaders joined two of the state interviews; one additional leader joined one-state interview. Demographic information was only collected from the primary participant from each state (Table 1). Participants were most often employed by their Comprehensive Cancer Control Program (83%) and held the title of director (40%), manager (37%), or coordinator (20%). A majority (60%) of participants had been in their current position for one to five years. Participants who were not in their current position when their state CCC plan was developed tended to discuss only current and future cancer control and prevention activities.

**Table 1:** State and interview participant characteristics

State characteristics	*n* = 30 (%)
High or low rural state designation, *n* (%)	
High	18 (60)
Low	12 (40)
Region, *n* (%)	
Northeast	4 (13)
South	9 (30)
Midwest	8 (27)
West	9 (30)

Interview participant characteristics	*n* = 30* (%)
Job affiliation	
Comprehensive cancer control program	25 (83)
Division of clinical preventive services	1 (3)
Chronic disease prevention section	1 (3)
Cancer collaborative	1 (3)
Prevention policy	1 (3)
Unknown	1 (3)
Job title (self-labeled), n* (%)	
Director	12 (40)
Manager	11 (37)
Coordinator	6 (20)
Unknown	1 (3)
Years of experience in current role, n* (%)	
> 1	2 (7)
1–2	6 (20)
3–5	12 (35)
6–10	2 (15)
> 10	2 (7)

*Two-state interviews had two additional participants and one-state interview had one additional participant (total *n*=35)

Only primary interviewee characteristics are displayed (*n*=30)

Results fell under three broad domains: (1) barriers to prioritizing rural populations in CCC plans; (2) successful strategies used to include a rural focus in current CCC plans; and (3) strategies that respondents suggested or planned to use in their next CCC plan to better address the needs of rural communities. To capture a range of how states have included rural foci in CCC plans, findings were synthesized across “high” and “low” rural states and geographic regions and are presented accordingly. Following the CDC guidelines on “Preferred Terms for Select Population Groups & Communities” [19] the term “stakeholder” is not included in this paper unless contained in a participant quotation.

### Barriers to incorporating a rural focus in cancer prevention and control plans

Participants from states whose CCC plans did not have a rural focus identified multiple reasons for the omission. Explanations centered around (1) beliefs that CCC plans should be broad and general, (2) difficulty defining “rural populations,” and (3) contending with long geographic distances (see Table 2).

**Table 2:** Summary of successful and planned strategies for states to incorporate a rural focus in cancer prevention and control plans

Strategy	Goal	Examples
Strategies that have been used in current plan		
Collaborating with healthcare systems in rural regions	To plan and implement rural community involvement	Recruited rural community members through existing collaborations with community health centers, local health departments, and Native nation clinics
Actively recruiting rural constituents	To promote representation of rural communities throughout the state	Formed regional cancer coalitions Held planning workgroups
Leveraging rural community–academic partnerships	To develop cross-sector relationships	Established academic researcher advisory group
Seeking Native nation involvement	To increase the diversity of the rural populations in the planning process	Recruited Native nation members to serve on cancer coalition committee
Strategies suggested for future CCC plans		
Building relationships with rural communities	To create systems that are accessible to rural communities throughout the state to participate in the planning process	Establish new relationships with rural health systems and community organizations
Seeking and inviting more rural engagement	To reflect the diversity of the rural populations in the planning process	Conduct stakeholder mapping Devote extra efforts to engage harder to reach groups
Developing a better understanding of the rural needs in their state	To better understand rural community needs and priorities	Conduct community needs assessment with a broad range of stakeholders at multiple levels Use secondary data to identify cancer health disparities and service gaps
Considering resources	To increase capacity of states to engage rural communities	Conduct community asset assessment Consider social determinants of health factors

#### Beliefs that CCC plans should be broad and general

Participants shared that they considered CCC plans to be general in nature and that the content should reflect the state as a whole: “*It’s a statewide plan, so it doesn’t focus on necessarily a particular population…it doesn’t break it down between city and the rest of the state; it’s the entire population. To be honest, we’ve never thought of breaking it down like that*.” Some participants also explained that their plans included an emphasis on cancer disparities overall rather than a focus on specific groups, particularly in states with multiple diverse populations: “*[The plan] doesn’t set any specific priorities for addressing specific disparities; it’s just kind of a general overview of disparities in cancer care*.” Additionally, some participants mentioned that their states’ population is primarily urban, with only a few small counties or areas that are considered rural. Therefore, a rural focus would not speak to the needs of their broader constituency.

#### Challenge of defining “rural population”

Some participants believed more rural partners should be involved in developing their state’s CCC plan, but they faced difficulties in defining their states’ rural population. For example, one participant said “*I was looking at the numbers… If you look at like HRSA…five counties are actually considered rural…[but] if I go by the state definition of rural with our collaborative members…about 31% [of our counties are rural].”* This respondent was referring to the variability in how federal and state statistical methodologies define “rural,” so the extent to which the plan should emphasize rurality depends on the definition being used. Participants also alluded to geographic diversity within counties, as a single county can encompass what feels, like rural areas as well as urban areas. Another respondent explained that in their CCC plan, “rural” falls under the umbrella term of “hard-to-reach and special populations” and is not specifically called out.

#### Geographic distance

Identifying and engaging partners in geographically remote areas was expressed as another obstacle. One participant noted that including perspectives from their state’s island inhabitants was challenging, since the islands can be sparsely populated and traveling across water is time-consuming. Another respondent indicated that because of distance, they have not yet met community leaders in rural areas of their state. A participant from a smaller state wondered if larger states with satellite offices in more rural areas had an easier time with outreach. Their state had only a handful of part-time employees, which limited their time and resources to connect with partners in rural areas: “*There’s only a certain number of paid people to sort of keep the energy going*.” Likewise, respondents perceived that rural partners face their own constraints related to distance, time, and capacity. For example, when in-person meetings are held in central parts of the state, they are inconvenient for partners from outlying areas to attend: “*For some it even required an overnight stay and depending on what their role with their employer was, it might not have been an activity that the employer would support their participation without them using their own time to do so.*” Participants explained that virtual meetings were not always the solution to overcoming geographic distance because some rural areas have poor broadband access. Finally, participants indicated that the disproportionate impact of COVID-19 on rural areas further exacerbated the three above-mentioned challenges: “*And of course, COVID hit and so that really just complicated everything, and as we know rural communities and partners got hit even harder.”*

### Strategies to incorporate a rural focus in CCC plans

Across states, four general successful strategies to incorporate a focus on rural needs in CCC plans were identified: (1) collaborating with healthcare systems in rural regions, (2) actively recruiting rural constituents, (3) leveraging rural community–academic partnerships, and (4) seeking Native nation involvement. Each strategy is described in detail below. It should be noted that some participants felt that a focus on rural cancer prevention and control was a *“given”* because their entire state is rural. They explained that the term ‘rural’ is not often used and is not a stated focus of their cancer plan because most of their population lives in rural areas. A participant shared, “*Honestly, our whole state is rural…So really any initiatives, anything that’s developed to address cancer within our state, is rural. Really just by definition*.” Other participants from rural states explained that even their urban centers were less populated compared to cities in more urban states.

#### Collaborating with healthcare systems in rural areas

Participants indicated that states leveraged connections with rural health care systems to facilitate input from rural residents when developing CCC plans. Through these relationships, partners from a variety of rural sites, including state hospitals and cancer centers, rural health associations, community health centers, local health departments, and Native nation clinics, assisted with planning. To include rural needs in the planning process, individuals from these sites were asked to *“participate as active members of the committees.”* For example, one participant explained that their state’s cancer consortium includes* “membership from some smaller cancer centers that are in more rural areas or serve rural communities.”*

#### Actively recruiting rural constituents

Respondents described how CCC plan developers appreciated the importance of including rural perspectives in their CCC plans. They identified rural constituents who understood the rural cancer prevention and control needs of their state and asked them to focus on those in the planning process. Plan developers sought rural constituent participation in several ways. For example, some formed regional cancer coalitions to represent rural areas of the state: *“There are regional coalitions specific to our cancer coalition. For example, [name] Southwest Regional Coalition*.*”* Participants described how they incorporated the *“rural voice”* through intentional planning workgroup composition: *“We did get the rural voice in these seven different work groups. We made sure it wasn’t metro voice only*.*”* One participant detailed that in their state they had meetings with representatives from various rural areas in which “*we presented data, which we had accumulated through our cancer registry…. and then they brought their needs and what they would like to see different*.*”* Across participants, many said either rural constituents were part of the planning process or meetings were held with rural constituents to ensure a focus on rural needs in the plan.

#### Leveraging rural community–academic partnerships

Respondents explained how they drew upon rural community–academic research partnerships already in place in order to understand rural health concerns. For example, one participant shared that a university in their state has a *“rural cancer advisory group that meets regularly, so we kind of pull from their talent and input*.*”* The interviewee and their colleagues would present ideas to the rural cancer advisory group and then incorporate their feedback into the CCC plan. Other participants described how they connected with advisory groups formed by researchers with rural expertise. They also sought input from these investigators, who had developed an understanding of rural needs over time through research collaborations with rural partners.

#### Seeking native nation involvement

Some participants indicated that their planning process involved active outreach to Native nations. They described how those cancer coalition members tended to share rural perspectives in addition to Native nation perspectives. For example, one participant spoke of an established partnership that their CCC plan developers have with a particular Native nation, which has been important in their planning process. “*We’ve always worked with them directly on the development of [our] plan, the activities related to their needs, and what they’re looking to accomplish*.*”* Another participant reflected that it takes time to build trust with Native nations, and the responsibility for that effort lies with the plan developers: “*there was a tribal member who came…I’d been trying to get ahold of her for years…I was very happy to see her there*.*”* Participants who talked about successful partnerships with Native nations also spoke of their intentionality and long-term commitment to building these partnerships.

### Planned strategies to incorporate a rural focus in the next CCC plan

When asked about the development of future CCC plans, participants indicated they are working on additional strategies to include a focus on rural health. They shared the impetus for why their next CCC plan would incorporate more of a rural emphasis. One participant explained that their intention to focus on rural communities in future is a result of “*slicing and dicing the data to understand better from…a race standpoint, socio-economic, rural/urban, frontier*.*”* Another participant indicated a need to have more *“synergy”* or *“synchronous timing”* between receiving recommendations from the CDC and enacting initiatives to meet CDC’s expectations regarding rural inclusion. One interviewee even shared that their CCC plan was called out by a keynote speaker at a cancer center meeting for not having a focus on health equity and disparities. Subsequently, they now have a “*more intentional focus right at the forefront to really include all disparities*.*”* Across participants, four general strategies were planned to support a rural focus in their next CCC plan: (1) building relationships with rural communities; (2) actively engaging rural partners in plan development; (3) developing a better understanding of the rural needs in their state; and (4) considering resources needed by rural residents and rural regions.

#### Building relationships with rural communities

Participants discussed the importance of having their CCC plan reflect community-identified needs and the critical role of rural communities in contributing to CCC plans: *“We can’t develop another cancer plan that hasn’t been vetted by not just, you know, academics and stakeholders, but by gatekeepers in the communities that we’re trying to affect*.*”* Participants recognized that rural community participation could be strengthened by developing better relationships; they discussed the need to *“develop those (rural community) relationships ahead of developing the plan*.*”* When speaking of relationships with rural communities, participants referred to a variety of individuals and organizations, including rural health systems, health departments, community organizations, and community leaders. Participants shared that they are working on building relationships now, because they believe that “*it takes a long time… to really understand the needs of these communities and to be able to successfully address some of the public health problems that they experience*.*”* Overall, there was a recognition of time needed for *“relationship building, trust building, and really starting to understand from their [rural residents’] perspective*.*”* Participants expressed hope that investments in building these relationships now could improve the extent to which future CCC plans address cancer prevention, screening, and treatment for rural populations.

#### Actively engaging rural partners in plan development

Respondents conveyed the ways in which they are seeking more rural community representation in the next round of CCC plan development. One participant explained that through a *“stakeholder mapping”* process, they examined which areas in their state were or were not represented in their CCC plan. As a result, they identified a need for more rural representation in the next CCC plan development process. Another participant described that in their state, they are being thoughtful about the composition of their next board as they are soliciting *“applications for new board members…we want someone from up north, from the east and we want it to be rural…We want diversity, geographic diversity in our new board members*.*”* Similarly, someone else talked about “*inviting, not just people that are already on the regional coalition, but others across the state from rural communities”* to cancer coalition meetings. In addition to inviting individuals to participate in planning, a participant indicated the importance of working with rural organizations and *“turning to those organizations that serve those populations.”*

#### Developing a better understanding of the rural needs in their state

Participants explained how they are working to better understand rural needs in their state. They talked about analyzing data and seeking perspectives from a broad range of rural partners, including researchers with rural expertise, clinicians who practice in rural areas, and rural residents. Participants shared that they are seeking information from multiple levels. As one participant said, they are pulling information from *“around the state, period…from all, all levels, from legislative to community faith-based, *etc*.”* A respondent spoke to the importance of listening to communities and “*trusting communities to know what they need for themselves… we should be thinking about it as, what are we missing in terms of not being able to serve them in the way that they’ve needed to be served?”* Finally, some participants were using data to better understand needs in rural areas. One participant explained that they looked at cancer incidence rates from across their state and compared them to available resources in given areas to identify unmet needs.

#### Considering resources needed by rural populations

Participants recognized that resources will be required to address the cancer-related needs of rural communities in their states’ next CCC plans. Transportation issues were commonly mentioned. One participant indicated that resources for transportation will have to be included in their next plan due to the impact of COVID-19: “*American Cancer Society stopped their Road to Recovery, their program for providing transportation, and that really hit rural populations*.*”* Another participant focused on long travel distances for rural residents and shared that they have created *“an extensive list of … transportation resources that are…ADA accessible.”* Another interviewee spoke to the need for financial resources as they discussed the *“dwindling economies”* in rural areas of their state, and how *“inadequate access to economic resources”* means that rural and urban residents have different reasons for not receiving cancer screenings. Several participants expressed concern about how they will secure screening resources for rural communities; according to one participant, *“the folks come in and get a screening, and it’s deemed they need to follow up with a colonoscopy, they don’t have transportation access, or a GI doc…we keep going back to the quandary of lack of resources, and, and that includes doctors, it includes insurance*.*”* One participant spoke of the need to get accurate information to rural residents because *"pockets of communities that are not as closely connected”* sometimes have cancer-related information that is *“really just kind of belief systems.”* Across respondents, needs related to social determinants of health (i.e., rural residents’ transportation, financial, healthcare, and information needs) were discussed as warranting support in their future CCC plans.

## Discussion

Our qualitative study illuminates how state cancer control leaders perceive rural populations as a priority in their CCC plans. We uncovered various reasons for a lack of focus on rural communities: the perception that plans should be broad and not population specific; the difficulties in defining exactly what “rural” means; and the challenges with engaging rural partners who live in distant, outlying areas of the state in plan development. We also found strategies that are being used, or will be used in future, to incorporate a rural focus in CCC plans. Respondents identified engagement of rural healthcare systems, constituents, and Native nation organizations as strategies imperative to enhancing rural inclusion. Finally, we found that participants planned to build more rural networks, engage more rural partners, develop a deeper understanding of rural needs, and identify needed resources in preparation for their next plan. This collective identification of the barriers and strategies describes ongoing needs and points to practical solutions for more rural inclusion in future CCC plans.

Unsurprisingly, we found that long travel distances and lack of broadband/internet availability and reliability were frequently cited inhibitors of engagement and involvement of rural partners. These factors prevent individuals living in rural areas from participating in in-person events and, sometimes, videoconference planning meetings. On a national level, studies have found that poor broadband access is particularly problematic in rural regions as compared to urban centers [20–22]. These factors highlight the importance of offering meetings in multiple locations across the state, with a hybrid design (in-person plus online) that encourages broad participation. Although effort is required to ensure greater geographic representation in CCC plan development, respondents were aware that rural partner engagement is crucial and have a desire to make improvements. In an analysis of the quality of 66 cancer plans using the Cancer Plan Index, Rochester and colleagues found the level of description of “global involvement of stakeholders” to be low [23]. Eliciting local perspectives can help garner buy-in, inclusion of rural-specific objectives, and subsequently, action, at the community level to support cancer prevention and control.

Some participants noted that their plan lacked rural inclusion because they prioritized broader health disparities or populations irrespective of location, rather than separately addressing rural disparities. Related factors identified by interviewees were challenges in applying different methodologies for determining which areas or counties “count” as rural or being in a state that is largely urban. Considering cancer disparities broadly masks the unique contributors to health inequities across different populations. It also hinders the development of successful targeted strategies to mitigate specific health disparities. The CDC’s Self-Assessment Tool specifies that reducing cancer disparities includes identifying the populations at greatest risk for cancer mortality [2], which in many states includes rural populations. Prior research identifies that cancer incidence and mortality are significantly higher for many types of cancer in rural counties than in non-rural or metropolitan counties across the U.S. [24, 25]. Developing a CCC plan with a statewide lens that ignores sociodemographic and geographic diversity related to population-level challenges and needs risks becoming inconsequential, particularly if the plan seeks to address health inequities. The Tool specifies that CCC plans should include plans to address cancer disparities. Thus, approaches are needed to address disparities among residents living in rural areas, which may be separate and distinct from effective approaches for residents living in urban centers. Implications of these findings extend beyond the need for states to enact strategies that will improve health for only rural residents. Specific cancer prevention and control strategies are needed for any state sub-population that is experiencing disparate outcomes (e.g., disparities by race or cancer type).

Participants shared a variety of strategies to increase rural engagement in future CCC plans. Recognizing that CCC plan leadership is often located in urban areas, the importance of creating and leveraging existing relationships with organizations in rural communities was highlighted. This corroborates a conclusion drawn by Allen et al. that increased bi-directional collaboration may help improve cancer control activities in rural communities [26]. Partnering organizations in rural areas may include direct healthcare service providers (e.g., hospitals, clinics), as well as coalitions, associations, and community-based organizations. For example, every state has a Human Resources and Services Administration (HRSA)-funded State Office of Rural Health that could partner in CCC plan development and implementation. County Extension offices funded by the U.S. Department of Agriculture are also potential partners as their staff often focus on health promotion activities in rural areas. Community health needs assessments are often conducted at the smallest level (county) and in some cases are conducted at regional, Native nation, territorial, and state levels. These needs assessments provide guidance to Community, Regional, or State Health Improvement Plans (e.g., CHIPs, RHIPs, SHIPs) [27] and could also be referenced at CCC planning meetings so developers can understand county-level contexts about locally identified needs, barriers, resources, and the lack thereof. Furthermore, CCC developers could leverage the expertise of faculty with rural health expertise and established partnerships at academic research centers. Community-engaged research demonstrates that programs that are responsive to problems identified and observed by communities and designed and executed in close collaboration with community partners are more effective than programs that simply review population-level data [28–30]. Importantly, participants recognized that building and sustaining such partnerships takes a significant investment of time and resources. It is important to bring together state cancer programs and coalitions to share best practices for engaging rural populations in the development of plans. For example, the CDC-funded Geographic Health Equity Alliance in collaboration with members of the CPCRN’s rural cancer workgroup offers a series of webinars to provide guidance on including rural and engaging rural communities in comprehensive control planning [31].

This research is not without limitations. Because we did not interview representatives from all 50 states, findings are not generalizable across the entire USA. Also, we asked only about current CCC plans and not all participants who completed interviews were involved in the development of their states’ CCC plans. Therefore, they were not able to provide detailed information about their state’s previous planning process. Interviews were conducted by investigators from nine centers; thus, inconsistencies across interviews may have occurred. However, it is also a strength that this study was conducted by a large national team, thus increasing the range of perspectives and experiences that guided our inquiry. The team was able to draw on their professional collaborations to successfully recruit CCC plan leadership to complete interviews from 30 states. This qualitative study is the first of its kind in that CCC plan leaders were directly engaged to understand how NCCCP recommendations for rural inclusion are implemented across the USA. Our results provide insights that may be used to develop strategies that significantly decrease rural cancer disparities across the USA.

It has been proposed that more diverse decision-making bodies make better decisions [32, 33]. When people from different backgrounds collaborate, individuals contribute their unique stories and perspectives, which can create new ideas and disrupt the status quo. Findings from this study highlight that for CCC plans to be effective statewide, needs and perspectives from the entire state must be included in the development process. To authentically include rural community voices in CCC plan development, creative and purposeful thought should be given to (1) the investment of time required for developing new or sustaining partnerships, (2) different resources that will be required to build trusted relationships, and (3) overcoming physical distance between teams and individuals engaged in the planning process.

## Supplementary Information

Below is the link to the electronic supplementary material.

Supplementary file1 (DOCX 19 KB)

Supplementary file2 (DOCX 18 KB)

## Data Availability

The datasets generated during and/or analyzed during the current study are not publicly available due to ongoing analyses but are available from the corresponding author on reasonable request.
