# Supplementary material for: Prioritizing rural populations in state comprehensive cancer control plans: a qualitative assessment
Source: Cancer Causes Control. 2023 Feb 25;34(Suppl 1):159–69. doi: 10.1007/s10552-023-01673-3 (PMC9959942; doi:10.1007/s10552-023-01673-3)
Supplement: Supplementary file 1 — Supplementary file1 (DOCX 19 KB) [file 10552_2023_1673_MOESM1_ESM.docx]

**Appendix: Standard Interview Guide**

INTRODUCTION

**Greeting:**

Hello, my name is ____________________ and I work with the [Name of University] and the Cancer Prevention and Control Research Network.  I really appreciate you taking the time to participate in this interview.

**Purpose:**

I am part of a team working on a CDC funded project to understand how comprehensive cancer control plans prioritize and facilitate cancer prevention and control activities for rural populations. We are particularly interested in how programs access and use cancer data for rural populations, the level of involvement of rural stakeholders in developing cancer control plans, and facilitators and barriers to inclusion of rural populations in cancer plans. **Information that you share with me will be combined with information from other cancer program directors to better understand how rural populations can be considered in cancer plans.**

**Process:**

I will ask you some specific questions, but the most important part of the discussion will be the information that you provide.  We value your thoughts, beliefs, and experiences and are grateful that you’re willing to share them with us. **My main goal is to learn from you and to have you feel comfortable during our conversation. Please know that we are coming to you as the expert in these matters and that there are no right or wrong answers to these questions.**

Our discussion today will last about 30-40 minutes. In thanks for your time and insight, you will receive a $30 gift card.

**Confidentiality:**

We will be recording our discussion, because we want to make sure we don’t miss anything you share with us. We want to assure you that all of your comments are confidential and that nothing you say will be connected with your name or shared with any of your colleagues. Participation in this study is completely voluntary and will not affect your employment in any way.

Do you have any questions or concerns for me before we begin?

Do we have your permission to start recording?

**Section 1: Establishing a context for the discussion to come (5 minutes)**

1. *Tell me about your professional background and current role(s) you play within your organization.*
2. *Tell me about your comprehensive cancer control program.*

a. *Where it is administratively housed?*

*b. How involved is your cancer coalition?*

**Section 2: CCC plan Development and Process (10 minutes)**

1. *Tell me about the last time your program developed its cancer plan.*
2. *Who were the stakeholders?*
3. *Were there stakeholders representing rural areas? (*Probes: HRSA-funded State Office of Rural Health leaders, Rural Health Coalition members, rural hospital administrators, etc.)
4. *What was the process like developing the cancer plan?*
5. *Who led the process? Please describe any workgroups/subcommittees that were responsible for different components (e.g., primary prevention, screening/early detection, etc.)?*
6. *What resources were available for the development of the plan?*
7. *Staff time? Funding? Did you receive technical assistance from the CDC or any other entity? If yes, what did the technical assistance consist of?*

**Section 3: Content of the Cancer Plan (5 minutes)**

1. *Tell me about the key priority areas and populations that are the focus of your plan.*

PROBE: IF RURAL IS MENTIONED: *Why was rural included as a focus?*

IF RURAL IS NOT MENTIONED: *What if anything, has prevented your program from including more of a rural focus in your cancer plans?*

**Section 4: Implementation of the Cancer Plan (5 minutes)**

1. *Tell me about the implementation of the cancer plan.*
2. *To what extent was the plan implemented?*
3. *Who implements it?*
4. *How were rural stakeholders involved in implementation, if at all?*

               PROBE: IF RURAL STAKEHOLDERS NOT INVOLVED: *What were the factors that prevented rural*

*stakeholders from being involved?*

**Section 5: Evaluation of the Cancer Plan (5 minutes)**

1. *Tell me about the evaluation of the cancer plan.*
2. *To what extent was the plan evaluated?*
3. *Who conducts the evaluation?*
4. *How were rural stakeholders involved in evaluation, if at all?*

PROBE: IF RURAL STAKEHOLDERS NOT INVOLVED: *What were the factors that prevented rural       stakeholders from being involved?*

**Section 6: Future Directions (5 minutes)**

1. *Thinking about the future, what are your ideas for the next cancer plan?*
2. *What will the process be?*
3. *What if any changes are you making to the process?*
4. *What do you see as some of the biggest potential priority areas for the next cancer plan?*
5. *Are there plans to include rural stakeholders?*

NOTE FOR INTERVIEWERS:  *if they are not thinking about rural…this may give some info about why not?)*

**Section 7: Closing (5 minutes)**

1. *What recommendations do you have for other program cancer plans about how to prioritize and better serve rural populations to address cancer disparities?*
2. Is there anything else that we did not cover that you would like to share with us?

Thank you for your time today and for your willingness to participate in this study.

Would you let us know your preferred way of receiving the gift card (email with a link or in the mail)? [If mail] Will you let me know your mailing address?

[TURN OFF AUDIO]
