# Supplementary material for: Prioritizing rural populations in state comprehensive cancer control plans: a qualitative assessment
Source: Cancer Causes Control. 2023 Feb 25;34(Suppl 1):159–69. doi: 10.1007/s10552-023-01673-3 (PMC9959942; doi:10.1007/s10552-023-01673-3)
Supplement: Supplementary file 2 — Supplementary file2 (DOCX 18 KB) [file 10552_2023_1673_MOESM2_ESM.docx]

**Appendix: Qualitative Analysis Codebook**

CPCRN Rural Cancer Workgroup Interview Codebook

(PIs: Askelson, Eberth, Yeager, Zahnd)

Assessing Rural Priorities and Stakeholders in State Cancer Control Plans: A Qualitative Study

V2 codebook: 08.02.2021

| **Code Concept** | **Abbreviation** | **Definition** |
| --- | --- | --- |
| **Section 1. Context and Background**  **“**Background” codes refer to background information about the participant as well as background information about the cancer plan development and process. | | |
| An individual’s professional Cancer Plan experience and responsibilities | **Background**  **Participant** | Comments from participants about their professional background and the role(s) they play in their current organization. Experience/years in Cancer Plan development. |
| The CCCP context | **Background**  **CCC Program** | Comments from participants about their comprehensive cancer control program, where it is administratively housed and the level of involvement of their cancer coalition or similar entities. |
| Cancer Plan details and logistics | **Background Plan Details** | Comments from participants about the year the last Cancer Plan was released, general scope, and frequency of being updated |
| **Section 2. CCC plan Development and Process**  “DEV” codes refer to the process and development of the latest Cancer Plan (CP) that participant discussed, who was involved in the development of the latest plan and what resources were used/needed during the development of the plan. | | |
| The process of developing the latest CP | **DEV_Process** | Comments from participants about what it was like during the development of the latest Cancer Plan and what consisted of the process. |
| Who was involved in developing the latest CP and what they did/did not do | **DEV_ Personnel** | Comments from participants about the personnel who developed the latest Cancer Plan. Note that this discussion may include references to individuals or partners who helped develop the cancer plan and/or groups or agencies responsible for rural or tribal areas.  Also use this code when participants discuss the personnel who **led** the process of developing the latest    Cancer Plan. Note that this discussion may be referring to workgroups or subcommittees and the different responsibilities these groups may or may not have had in the process.  Also use this code when participants discuss any personnel/stakeholders or partners who were not represented or should have been involved in developing the Cancer Plan but were not. |
| Resources used/needed in developing the latest CP | **DEV_Resources** | Comments from participants about what resources were available for the development of the latest Cancer Plan, including staff time, funding, TA from the CDC or other agencies, or any other discussed resources. Also use this code if participants talk about resources that were not available or they wish had been available during the development of the Cancer Plan. |
| Level of rural/tribal involvement in developing latest CP | **DEV_Rural/Tribal Involvement** | Comments about the extent to which rural and/or tribal areas were included as a focus or priority area in the latest Cancer Plan and the factors that prevented or assisted rural and/or tribal areas from being a priority area in the plan. |
| **Section 3. Content of the Cancer Plan**  “CON” codes refer to participant comments about the key content or priority areas in the latest Cancer Plan and their perceptions on the level of focus rural and tribal areas received in the plan. | | |
| Key content or priority areas in the latest CP | **CON_Priority Areas** | Comments from participants describing or explaining the key content or priority areas in the latest Cancer Plan. |
| **Section 4. Implementation of the Cancer Plan**  “IMP” codes refer to participants’ experiences with the implementation of the latest Cancer Plan and their perceptions on the level of rural and tribal stakeholder involvement in implementing the plan. | | |
| Implementation process for latest CP | **IMP_Process** | Comments from participants about their experiences implementing the latest Cancer Plan, including the extent to which the plan was or was not implemented and who participated in implementing the plan. |
| Level of rural/tribal involvement in implementing latest CP | **IMP_Rural/Tribal Involvement** | Comments about to what extent rural and/or tribal stakeholders were involved in the implementation of the latest Cancer Plan and the factors that prevented or assisted rural and/or tribal areas from being involved in implementing the plan. |
|  |  |  |
| **Section 5. Evaluation of the Cancer Plan**  “EVAL” codes refer to participants’ experiences with the evaluation of the latest Cancer Plan and their perceptions on the level of rural and tribal stakeholder involvement in evaluating the plan. | | |
| Evaluation process for latest CP | **EVAL_Process** | Comments from participants about their experiences evaluating the latest Cancer Plan, including the extent to which the plan was or was not evaluated and who participated in evaluating the plan. |
| Level of rural/tribal involvement in evaluating latest CP | **EVAL_Rural/Tribal Involvement** | Comments about to what extent rural and tribal stakeholders were involved in the evaluation of the latest Cancer Plan and the factors that prevented or assisted rural and/or tribal areas from being involved in evaluating the plan. |
| **Section 6. Future Directions**  “FUT” codes refer to participants’ thoughts and opinions about the future direction for the development and dissemination/evaluation of the next Cancer Plan, any changes they foresee making to the process or to the plan itself, priority areas as they see them for the next plan, and any recommendations or lessons learned to be applied to future Cancer Plans. | | |
| Next CP development process and key priority areas to be targeted | **FUT_Process and Development** | Comments from participants about their thoughts and opinions on the next Cancer Plan that will be developed and how they foresee the process unfolding for that future plan.  Also, include any comments about what participants foresee as the biggest/most important priority areas for the **next** Cancer Plan. |
| Level of rural/tribal stakeholder involvement planned or needed for future CP | **FUT_Stakeholders** | Comments from participants on what extent future CCC plans will include rural and/or tribal stakeholders. |
| Recommendations or lessons learned to be applied to the future development of a CP | **FUT_Recomendations** | Comments about how and what participants felt was important for other programs/states to know in order to be successful prioritizing, including and serving rural and/or tribal populations in the process of developing a Cancer Plan. This includes any comments about things to emphasize or avoid in future next steps or suggestions/lessons learned that can be applied to the development of the next plan. |
| Responses to questions that are not part of the guide but can be coded for future use TBD | **Miscellaneous** | Responses to inquiries from interviewers about topics not on the interview guide. (e.g., how does Internet service in rural areas compare to urban areas? OR How does your organization or HD tend to identify or define what a rural area is, vs. an urban or suburban area? |
